# Breed, age and gender distribution of dogs with chronic hepatitis in the United Kingdom

**DOI:** 10.1016/j.tvjl.2011.11.024

**Published:** 2012-07

**Authors:** N.H. Bexfield, R.J. Buxton, T.J. Vicek, M.J. Day, S.M. Bailey, S.P. Haugland, L.R. Morrison, R.W. Else, F. Constantino-Casas, P.J. Watson

**Affiliations:** a Department of Veterinary Medicine, University of Cambridge, Cambridge, UK; b Finn Pathologists, Diss, Norfolk, UK; c School of Veterinary Sciences, University of Bristol, Bristol, UK; d The Royal Veterinary College, University of London, Hatfield, UK; e Rest Associates, Swaffham Prior, Cambridge, UK; f The Veterinary Pathology Unit, University of Edinburgh, Edinburgh, UK

**Keywords:** Dog, Liver, Hepatitis, Breed representation

## Abstract

Standardised histological criteria are now available for the diagnosis of canine chronic hepatitis (CH). CH is common in dogs, but no studies have reported breed, age and gender distributions in the United Kingdom (UK). The objective of this study was to determine which breeds had an increased risk for developing CH in the UK and to report the age and gender distribution for those breeds. The databases of six veterinary histopathology laboratories were searched for cases with a histological diagnosis of CH according to standardised criteria. The breed, age and gender of dogs was recorded and compared to a control population to calculate the odds ratio and 95% confidence intervals for developing CH.

A total of 551 cases of CH were identified, consisting of 61 breeds. Nineteen breeds were represented by five or more cases. Breeds with an increased risk for developing CH included the American cocker spaniel, Cairn terrier, Dalmatian, Dobermann pinscher, English cocker spaniel, English springer spaniel, Great Dane, Labrador retriever and Samoyed. The median age at diagnosis for all breeds with CH was 8 years (range 7 months to 16 years). Dalmatians, Dobermann pinschers and English springer spaniels with CH were significantly younger than Cairn terriers, English cocker spaniels and Labrador retrievers with CH. Females were over-represented when all cases were examined together. In conclusion, several breeds in the UK have an increased risk of CH, some of which have not been previously reported.

## Introduction

Canine chronic hepatitis (CH) is common in the United Kingdom (UK), with a reported postmortem prevalence of 12% in a first opinion practice setting (Watson et al., 2010). Clinical signs, clinicopathological findings and results of diagnostic imaging are non-specific. Histological examination of liver is required for a definitive diagnosis. The histological criteria for a diagnosis of canine CH recently have been standardised by the World Small Animal Veterinary Association (WSAVA) and include the presence of hepatocellular apoptosis or necrosis, a variable mononuclear or mixed inflammatory cell infiltrate, regeneration and fibrosis (Van den Ingh et al., 2006).

The aetiology of most cases of canine CH remains unknown (Poldervaart et al., 2009). Known causes identified in a small proportion of cases include the virus canine adenovirus (Chouinard et al., 1998), bacteria including leptospires (Bishop et al., 1979) and *Helicobacter* spp. (Boomkens et al., 2005), and several toxins and drugs (Bunch, 1993). Defects in copper metabolism have also been described in several breeds, including the Bedlington terrier (Twedt et al., 1979; van De Sluis et al., 2002), Dalmatian (Webb et al., 2002), Dobermann pinscher (Mandigers et al., 2004), Labrador retriever (Hoffmann et al., 2006), Skye terrier (Haywood et al., 1988) and West Highland white terrier (Thornburg et al., 1996). α-1 Antitrypsin deficiency has been linked to CH in the English cocker spaniel (Sevelius et al., 1994). Immune-mediated disease is suspected in some dogs, particularly Dobermann pinschers, but so far studies have failed to conclusively demonstrate a primary immune-mediated aetiology (Andersson and Sevelius, 1992; Weiss et al., 1995; Dyggve et al., 2011).

A previous study has reported breed, age and gender distributions to CH in Swedish dogs (Andersson and Sevelius, 1991), although this study was performed two decades ago and prior to the establishment of standardised histological criteria for diagnosis of CH. Breeds with an increased risk for CH in that study included the American and English cocker spaniel, West Highland white terrier, Labrador retriever, Dobermann pinscher and Scottish terrier. The disease was more common in middle aged to older animals and there was a gender predisposition in male English and American cocker spaniels and female Labrador retrievers.

Dog breeds at risk for developing CH may change with time and geographic location due to genetic and environmental factors. The aim of the present study was to identify dog breeds with an increased risk for developing CH in the UK using recently standardised criteria for histological diagnosis. We also aimed to report the gender and age of at-risk breeds.

## Materials and methods

### Study population

The databases of six veterinary histopathology laboratories (Universities of Bristol, Cambridge and Edinburgh, Finn Pathologists, Rest Associates and the Royal Veterinary College, RVC) were searched for cases with a histological diagnosis of CH. Databases were searched from 2001–2008 (Bristol), 2001–2008 (Cambridge), 2001–2008 (Edinburgh), 2001–2008 (Finn Pathologists), 2006–2008 (Rest Associates) and 2001–2008 (RVC). Criteria for a histological diagnosis of CH had to include the presence of all of the following: mononuclear or mixed inflammatory cell infiltration, hepatocyte necrosis or apoptosis, fibrosis and regeneration (Van den Ingh et al., 2006). In all institutions, at least one board qualified pathologist reviewed either the histopathology slides (502 cases) or histopathology report (49 cases).

Although we included cases that were biopsied prior to the establishment of WSAVA criteria for a diagnosis of CH, in all cases histopathology slides or the histopathology report were reviewed after the publication of these criteria to confirm that these criteria were met. Rhodanine stained sections from cases of CH identified from the Cambridge histopathology database were also evaluated for the presence of copper. If rhodanine sections were not available, where possible, additional slides were cut from archived tissues and stained. Copper accumulation was scored on sections with rhodanine stain as 0 (absence or rare copper positive cells), 1 (few random copper positive cells), 2 (moderate numbers of copper positive cells) and 3 (many copper positive cells in all zones) using a previously established semi-quantitative scoring system (Shih et al., 2007).

Data collected from the histopathological records included breed, age and gender. To identify if an individual breed had an increased risk of CH, a UK-based control population consisting of dogs registered with a microchip company (Petlog) was used for comparison. The control population consisted of 175,442 and 311,085 dogs from 2001 and 2008, respectively, to reflect the time period over which cases were collected. Only breed information was available for the control population.

### Statistical analysis

Statistical analysis was performed using commercially available statistical software (PASW Statistics v1). Odds ratios (ORs) and the 95% confidence intervals (CIs) were calculated for all breeds with five or more cases of CH using both the 2001 and 2008 control data. An OR was considered to be significantly increased only if the OR and 95% CI were >1 using both the control data from 2001 and 2008. Age data are presented as median ± range. Differences in age between breeds with CH were tested using the Mann–Whitney test. The gender ratio of individual breeds with CH was compared with the gender ratio of all dogs with CH. As the gender of the control population was not known, it was not possible to calculate the statistical significance of any apparent gender over-representations in certain breeds. *P *< 0.05 was considered to be statistically significant.

## Results

A total of 551 cases of canine CH were identified. The number of cases per histopathology laboratory was 26 (Bristol), 130 (Cambridge), 23 (Edinburgh), 327 (Finn Pathologists), 19 (Rest Associates) and 26 (RVC); 90% of cases submitted to Finn Pathologists and 78% of cases submitted to Cambridge originated from first opinion clinics, with the remainder originating from referral practices. Data was not available to indicate the percentage of cases submitted by first opinion practices to the other histopathology laboratories. Eight cases were excluded because the breed was unknown. Sixty-one breeds were represented and 19 were represented by five or more cases and were included in the analysis (Table 1). The American cocker spaniel, Cairn terrier, Dalmatian, Dobermann pinscher, English cocker spaniel, English springer spaniel, Great Dane, Labrador retriever and Samoyed were found to be at increased risk for developing CH (Table 1).

Age was known for 521 cases. The median age for all breeds with CH combined was 8 years (range 7 months to 16 years). The median age and range for breeds at an increased risk for developing CH are given in Table 2. Dalmatians, Dobermann pinschers and English springer spaniels with CH were significantly younger than Labrador retrievers (*P *< 0.001), English cocker spaniels (*P *< 0.001) and Cairn terriers (*P *< 0.0001) with CH. American cocker spaniels were significantly younger than English cocker spaniels with CH (*P *< 0.05). There was no significant difference in age between Labrador retrievers and English cocker spaniels, Cairn terriers and Samoyed or between English springer spaniels, Dalmatians, Dobermann pinschers and Great Danes with CH.

Gender was known for 540 cases. There were 326 females and 214 males and the female to male ratio 1.5:1.0. When individual breeds were examined, female Dalmatians (9 F, 1 M), Dobermann pinschers (16 F, 8 M), English cocker spaniels (34 F, 19 M), English springer spaniels (60 F, 20 M) and Labrador retrievers (63 F, 32 M) appeared to be over-represented. Male American cocker spaniels also appeared to be over-represented (1 F, 5 M). No apparent gender over-representation was seen in the Cairn terrier, Great Dane and Samoyed.

Out of the 130 cases identified from the Cambridge database, rhodanine stained sections were available from 110. Copper accumulation was scored as 0 (*n *= 81), 1 (*n *= 22), 2 (*n *= 3) and 3 (*n *= 4). Breeds with copper scores of 2 included the Dobermann pinscher (*n *= 1), English cocker spaniel (*n *= 1), and Jack Russell terrier (*n *= 1). The distribution of copper in all dogs with a score of 2 was multifocal and excess copper was located primarily in the peri-portal region. Breeds with copper scores of 3 included the Bedlington terrier (*n *= 2), Dalmatian (*n *= 1) and Dobermann pinscher (*n *= 1).

## Discussion

This is the first study to document breeds at an increased risk for developing canine CH in the UK and to provide age and gender data for individual breeds. The only other study that compared dogs with CH to a control population was performed two decades ago on a population of Swedish dogs and using non-standardised criteria for diagnosis of CH (Andersson and Sevelius, 1991). Both the previous and the present study demonstrated that the American and English cocker spaniel, Dobermann pinscher and Labrador retriever are at an increased risk for developing CH, but the present study documents additional at risk breeds, including the Cairn terrier, English springer spaniel, Great Dane and Samoyed.

The median age at diagnosis was 8 years, with a range from 7 months to 16 years. This compares to previous studies which report median ages at diagnosis of 7.7 years (Poldervaart et al., 2009) and 5.9 years (Andersson and Sevelius, 1991). Females were predisposed to CH when all dogs were considered together, again consistent with previous reports (Andersson and Sevelius, 1991; Poldervaart et al., 2009). When individual breeds were examined, female Dalmatians, Dobermann pinschers, English cocker spaniels, English springer spaniels and Labrador retrievers appeared to be over-represented. Male American cocker spaniels also appeared to be over-represented, but no apparent gender over-representation was demonstrated in the Cairn terrier, Great Dane or Samoyed, although numbers of affected animals were small. As the gender of the control population was not known, the significance of these apparent gender over-representations could not be statistically evaluated.

The Dobermann pinscher had one of the highest odds of developing CH and the disease is well recognised in this breed (Johnson et al., 1982; Crawford et al., 1985; Strombeck et al., 1988; Thornburg, 1998; Speeti et al., 2003; Dyggve et al., 2011). In the present study, the median age at diagnosis was 5 years and 4 months, which compares to a median of 5 years reported previously (Crawford et al., 1985). There was no significant difference in age between Dobermann pinschers, Dalmatians, English springer spaniels and Great Danes with CH. An apparent female over-representation to the disease was observed, consistent with previous studies (Johnson et al., 1982; Crawford et al., 1985).

A recent study documents a dog leucocyte antigen haplotype association in affected dogs (Dyggve et al., 2011) suggesting a possible role for the immune system in the development of CH. The apparent female over-representation in the Dobermann pinscher and other breeds, may support the presence of an immune-mediated aetiology, similar to a possible female predisposition to other immune-mediated diseases (Reimer et al., 1999). Although studies have demonstrated antinuclear and anti-liver membrane protein autoantibodies (Andersson and Sevelius, 1992; Weiss et al., 1995) or cell-mediated immunity to liver membrane protein in a variety of breeds with CH (Poitout et al., 1997), these studies were unable to determine if this was a primary cause or a secondary phenomenon.

Although the aetiology of CH is unknown in the majority of cases (Poldervaart et al., 2009), certain breed-specific aetiologies do exist. Excess copper is one of the most widely implicated aetiological agents, with a copper-associated hepatopathy reported in the Bedlington terrier (Twedt et al., 1979; van De Sluis et al., 2002), Dalmatian (Webb et al., 2002), Dobermann pinscher (Mandigers et al., 2004), Labrador retriever (Hoffmann et al., 2006), Skye terrier (Haywood et al., 1988) and West Highland white terrier (Thornburg et al., 1996).

A recent study in the Netherlands demonstrated that 36% of cases of primary hepatitis had increased copper accumulation on rubeanic stained sections of liver tissue (Poldervaart et al., 2009). Out of the 110 Cambridge cases which were histochemically stained for copper in the present study, seven (6%) dogs had a copper score of 2 or 3. Copper scores >2 are considered to be abnormal and are suggestive of an aetiopathogenetic role for copper in the development of CH (Shih et al., 2007). In our cohort of dogs, it appears that copper does not play a significant role in the aetiology of CH in dogs in the UK, although larger studies are required to confirm this finding. From the results of previous studies, significant amounts of copper were not present in the livers of English springer spaniels (Bexfield et al., 2011) or Labrador retrievers (House et al., 2008) with CH in the UK on the basis of histopathological examination.

The English springer spaniel had an increased risk for developing CH, and had the second highest number of affected dogs overall. Females appeared to be predisposed and the median age at diagnosis was 5 years. Although this is the first report of an apparent breed predisposition to CH in the English springer spaniel, the authors had already been made aware of an apparent recent increase in disease prevalence in the breed in the UK and a clinical description of the disease has been published (Bexfield et al., 2011). In this latter report, females were again predisposed although the median age at diagnosis was 3 years 4 months. The aetiology of CH in English springer spaniels in the UK is also unknown.

The Labrador retriever was the breed most frequently diagnosed with CH, and was at an increased risk for developing CH. The median age at diagnosis was 8 years 3 months, which compares to 9 years 3 months previously reported (Shih et al., 2007). As with our study, some previous studies, report an apparent female over-representation to CH in the Labrador retriever (Hoffmann et al., 2006; Smedley et al., 2009), although this was not demonstrated in the study by Shih et al. (2007).

English cocker spaniels were found to be at increased risk for developing CH. A previous study reported that male English cocker spaniels were predisposed to CH (Andersson and Sevelius, 1991), but females outnumbered males in the present study (34 female, 19 male). The English cocker spaniel was one of the breeds in which CH occurred at an older age (median age 8 years 9 months). English cocker spaniels with CH have been shown to accumulate two types of α-1 antitrypsin in their hepatocytes (Sevelius et al., 1994), although reduced serum α-1 antitrypsin, a hallmark of human α-1 antitrypsin deficiency, is not present (Sevelius et al., 1994).

Dalmatians also showed an increased risk for developing CH and females were apparently over-represented. CH has been reported in the Dalmatian previously (Napier, 1996; Webb et al., 2002). This is the first study to document an increased risk for developing CH in the Cairn terrier, Great Dane and Samoyed. The Cairn terrier and Samoyed were the breeds diagnosed with CH at the oldest ages, with a median age at diagnosis of 10 years 2 months and 10 years, respectively. There was no apparent gender over-representation in any of these three breeds, although numbers of affected animals were small.

Despite previous reports of a breed association in the West Highland white terrier (Andersson and Sevelius, 1991; Thornburg et al., 1996), this breed did not have an increased risk for developing CH in our study. The Scottish terrier has also been reported to be predisposed to the development of CH (Andersson and Sevelius, 1991), but only two cases were identified in this study.

The present study has several limitations. The control population consisted of dogs registered with a microchip company and may be subject to biases and so not reflect the general dog population in the UK. As some samples submitted to histopathological laboratories were from referral rather than first opinion practices, the use of a separate control group for these representing the referred population of dogs would have been of value. Previous studies examining breed distributions in different diseases have used smaller control populations, such as animals registered with an insurance company (Davison et al., 2005), the referred hospital population (Hess et al., 2000) or the Swedish Kennel Club (Andersson and Sevelius, 1991). We used control data from both 2001 and 2008 to span the period over which histological data was collected and to take into account possible changes in breed proportions over this period. Identical breed predispositions were found when data from 2001 and 2008 was analysed.

The clinical history of our control population was not known and it is possible that some dogs in this group had CH. No details of the age or gender of the control population were available and it is possible that an age and gender bias may have existed, which could influence the results of apparent age and gender predispositions to CH. It is also possible that the breed name on the histopathology report might not accurately reflect the true breed identity, leading to potential errors in conclusions drawn regarding breed predispositions.

Although a review of the histopathological slides using standardised WSAVA criteria was performed in the majority of cases, masked assessment of all slides by one or more histopathologists should ideally be performed in future studies. Although we included cases which were biopsied prior to the establishment of WSAVA criteria for a diagnosis of CH, in all cases, histopathology slides or the histopathology report were reviewed to confirm that these criteria were met.

## Conclusions

This study demonstrates that several breeds of dog in the UK are at increased risk for developing CH. This is the first report to document an apparent increased risk of CH in the Cairn terrier, English springer spaniel, Great Dane and Samoyed. When all dogs with CH were considered together, the median age at diagnosis was 8 years and females appeared to be over-represented. Specific age and gender predispositions exist for individual breeds.

## Conflict of interest statement

None of the authors of this paper has a financial or personal relationship with other people or organisations that could inappropriately influence or bias the content of the paper.

## Figures and Tables

**Table 1:** Breeds with canine chronic hepatitis (CH) represented by more than five cases.

Breed	Number of cases of CH	2001 Control population				2008 Control population			
		Number of control dogs	Odds ratio	95% Confidence interval	*P* value	Number of control dogs	Odds ratio	95% Confidence interval	*P* value
**American cocker spaniel**	6	194	10.1	4.6–22.4	<0.001	161	21.6	9.7–47.9	<0.001
Border Collie	17	8777	0.6	0.4–1.0	0.048	13,444	0.7	0.4–1.2	0.20
**Cairn terrier**	9	988	2.9	1.5–5.6	0.005	1441	3.6	1.9–6.9	0.001
Cavalier King Charles spaniel	9	3212	0.9	0.5–1.7	0.874	7618	0.7	0.4–1.3	0.27
Crossbred	30	42,624	0.1	0.1–0.3	<0.001	57,759	0.3	0.2–0.4	<0.001
**Dalmatian**	10	1285	2.5	1.4–4.7	0.008	1406	4.1	2.2–7.7	<0.001
**Dobermann pinscher**	24	1163	6.9	4.6–10.4	<0.001	1250	11.5	7.6–17.3	<0.001
**English cocker spaniel**	53	4720	4.3	3.3–5.8	<0.001	13,466	2.4	1.8–3.2	<0.001
**English springer spaniel**	80	3176	9.4	7.4–11.9	<0.001	9779	5.3	4.2–6.7	<0.001
Golden retriever	8	4917	0.5	0.3–1.0	0.066	5835	0.8	0.4–1.6	0.63
**Great Dane**	6	617	3.2	1.5–7.0	0.014	856	4.0	1.9–8.9	0.004
German Shepherd dog	15	9343	0.5	0.3–0.8	0.005	10,854	0.8	0.5–1.3	0.41
Jack Russell terrier	19	8994	0.7	0.4–1.1	0.096	23,523	0.4	0.3–0.7	<0.001
**Labrador retriever**	95	15,794	2.1	1.7–2.7	<0.001	29,612	2.0	1.6–2.5	<0.001
Rottweiler	5	2800	0.6	0.2–1.3	0.299	4525	0.6	0.3–1.5	0.37
**Samoyed**	5	375	4.3	1.8–10.1	0.007	229	12.6	5.3–29.9	<0.001
Staffordshire Bull terrier	5	9123	0.2	0.1–0.4	<0.001	22,066	0.1	0.1–0.3	<0.001
West Highland white terrier	20	5641	1.2	0.7–1.8	0.54	8102	1.4	0.9–2.2	0.135
Yorkshire terrier	15	5782	0.9	0.5–1.5	0.81	7654	1.1	0.7–1.9	0.650

Breeds in bold font are those at an increased risk for developing CH. M, male; F, female.

**Table 2:** Age and gender data for breeds with an increased risk for developing canine chronic hepatitis (CH).

Breed	Median age at diagnosis (range)	Gender
American cocker spaniel	5 years 6 months (2 years to 11 years 3 months)	1 F, 5 M
Cairn terrier	10 years 2 months (7 years to 13 years 5 months)	4 F, 5 M
Dalmatian	4 years 7 months (3 years to 12 years)	9 F, 1 M
Dobermann pinscher	5 years 4 months (2 years 6 months to 10 years)	16 F, 8 M
English cocker spaniel	8 years 9 months (1 year 3 months to 14 years)	34 F, 19 M
English springer spaniel	5 years (1 year 2 months to 11 years)	60 F, 20 M
Great Dane	6 years 2 months (1 year 2 months to 7 years 11 months)	3 F, 3 M
Labrador retriever	8 years 3 months (2 years 8 months to 13 years)	63 F, 32 M
Samoyed	10 years (3 years 1 month to 11 years)	2 F, 3 M

M, male; F, female.
